# Hemoglobin induces inflammation after preterm intraventricular hemorrhage by methemoglobin formation

**DOI:** 10.1186/1742-2094-10-100

**Published:** 2013-08-06

**Authors:** Magnus Gram, Snjolaug Sveinsdottir, Karsten Ruscher, Stefan R Hansson, Magnus Cinthio, Bo Åkerström, David Ley

**Affiliations:** 1 Division of Infection Medicine, Lund University, S-221 84 Lund, Sweden; 2 Department of Pediatrics, Lund University, S-221 84 Lund, Sweden; 3 Division of Neurosurgery, Laboratory for Experimental Brain Research, Lund University, S-221 84 Lund, Sweden; 4 Department of Obstetrics & Gynecology, Lund University, S-221 85 Lund, Sweden; 5 Department of Electrical Measurements, Lund University, S-221 84 Lund, Sweden

**Keywords:** Hemoglobin, Intraventricular hemorrhage, Preterm birth, Perinatal brain damage, Astrocyte, Inflammation, Cerebrospinal fluid, Periventricular brain tissue

## Abstract

**Background:**

Cerebral intraventricular hemorrhage (IVH) is a major cause of severe neurodevelopmental impairment in preterm infants. To date, no therapy is available that prevents infants from developing serious neurological disability following IVH. Thus, to develop treatment strategies for IVH, it is essential to characterize the initial sequence of molecular events that leads to brain damage. In this study, we investigated extracellular hemoglobin (Hb) as a causal initiator of inflammation in preterm IVH.

**Methods:**

Using a preterm rabbit pup model, we investigated the molecular mechanisms and events following IVH. We also characterized the concentrations of cell-free Hb metabolites and pro-inflammatory mediators in the cerebrospinal fluid (CSF) of preterm human infants and rabbit pups. Finally, Hb metabolites were evaluated as causal initiators of inflammation in primary rabbit astrocyte cell cultures.

**Results:**

Following IVH in preterm rabbit pups, the intraventricular CSF concentration of cell-free methemoglobin (metHb) increased from 24 to 72 hours and was strongly correlated with the concentration of TNFα at 72 hours (r^2^ = 0.896, *P* <0.001). Also, the mRNA expression of TNFα, IL-1β, and Toll-like receptor-4 and TNFα protein levels were significantly increased in periventricular tissue at 72 hours, which was accompanied by extensive astrocyte activation (that is, glial fibrillary acidic protein (GFAP)staining). Furthermore, exposure of primary rabbit astrocyte cell cultures to metHb caused a dose-dependent increase in TNFα mRNA and protein levels, which was not observed following exposure to oxyhemoglobin (oxyHb) or hemin. Finally, a positive correlation (r^2^ = 0.237, *P* <0.03) between metHb and TNFα concentrations was observed in the CSF of preterm human infants following IVH.

**Conclusions:**

Following preterm IVH, increased metHb formation in the intraventricular space induces expression of pro-inflammatory cytokines. Thus, the formation of metHb might be a crucial initial event in the development of brain damage following preterm IVH. Accordingly, removal, scavenging, or neutralization of Hb could present a therapeutic opportunity and plausible approach to decreasing the damage in the immature brain following preterm IVH.

## Background

Severe cerebral intraventricular hemorrhage (IVH) in preterm infants continues to be a major clinical problem, occurring in about 15% to 20% of very preterm infants [1,2] with an associated neonatal mortality of 20% to 50%. More than 50% of surviving infants develop post-hemorrhagic ventricular dilatation and 40% to 80% develop severe neurological impairment, mainly cerebral palsy and intellectual disability [3-7]. To date, no therapy is known to prevent infants from developing serious neurological disability following IVH.

Despite substantial research over the years, the complex initial molecular mechanisms following IVH and subsequently causing brain injury are incompletely understood. Previous studies investigating periventricular white matter brain damage in the immature brain have largely focused on hypoxia–ischemia and infection–inflammation as primary events [8]. Findings show that exogenous and endogenous activators of innate immunity cause astrogliosis and microglial activation. The resulting pro-inflammatory response and generation of reactive oxygen species (ROS) cause cell death and/or maturational arrest in the vulnerable pre-oligodendrocyte population [9]. IVH in the immature brain is also followed by microglial and astrocytic activation and increased expression of the pro-inflammatory cytokines TNFα and IL-1β in periventricular brain tissue [10]. Indeed, binding of TNFα to the TNF receptor (TNFR) 1 has been described as a pivotal upstream event in the induction of intracellular pathways leading to apoptotic and necrotic cell death [11]. Intervention aimed at blocking the effect of TNFα following IVH has been associated with decreased periventricular cell death [12].

In preterm infants with IVH, rupture of the germinal matrix vasculature and the ventricular ependyma leads to the deposit of extravasated blood in the intraventricular cerebrospinal fluid (CSF), and the subsequent hemolysis leads to high concentrations of extracellular hemoglobin (Hb) in the intraventricular space. OxyHb (that is, ferrous (Fe^2+^) Hb) can undergo spontaneous auto-oxidation in which metHb (that is, ferric (Fe^3+^) Hb) and superoxide are formed. Downstream reactions lead to the formation of ferryl (Fe^4+^) Hb, ROS, and free heme. The subsequent degradation of heme results in the generation of bilirubin, carbon monoxide and free iron [13]. Cell-free Hb has been proposed as an initiator of pro-inflammation, chemotaxis and necrosis/apoptosis in intracranial hemorrhage (ICH) [14,15]. Studies have indicated that metabolites of extracellular Hb have pro-inflammatory effects in microglia, endothelial cells and macrophages and, indeed, may behave as activators of innate immunity, that is, as ligands of the Toll-like receptor (TLR) system [16-18]. However, the release and metabolism of extracellular Hb and their correlation with inflammation following cerebral hemorrhage have, to our knowledge, not been characterized.

In this study, we hypothesized that after preterm IVH, extracellular Hb metabolites act as causal initiators of inflammation, thereby constituting a critical upstream event that leads to periventricular cell death. We investigated this hypothesis *in vivo* using the preterm rabbit pup model. This model is well suited for the study of molecular mechanisms and events of IVH because preterm rabbit pups have a germinal matrix, develop spontaneous IVH [19] and exhibit brain maturation corresponding to that of a human infant at 28 to 30 weeks of gestation [20]. In the rabbit pup model, intraperitoneal injection of glycerol causes intracranial hypotension and an increased transmural pressure gradient, which predisposes to rupture of the fragile germinal matrix capillaries leading to IVH. This closely resembles the origin of IVH in preterm children, considered to be caused by germinal matrix vessel rupture following intracranial blood pressure fluctuations. Furthermore, the hemorrhage is confined to the intraventricular space and results in a progressive ventricular enlargement very similar to that seen in preterm infants following IVH. Other small animal models of IVH have utilized injection of autologous blood into the ventricles but in these models the characteristic periventricular brain damage with resulting neurobehavioral deficits as well as the development of posthemorrhagic ventricular dilatation is not observed [21]. Application of high-frequency ultrasound (HFU) in this animal model enables precise assessment of ventricular dilatation and accurate *in vivo* sampling of CSF from the intraventricular space [22].

We observed an accumulation of extracellular metHb in intraventricular CSF during the first 72 hours after IVH and a resulting high correlation with intraventricular levels of TNFα. In primary astrocyte cell culture, extracellular metHb, as opposed to other Hb metabolites, induced increased TNFα mRNA and protein expression. Finally, we demonstrated a positive relationship between extracellular metHb and TNFα in the CSF of preterm human infants following IVH.

## Methods

### Animals

**Figure 1:**
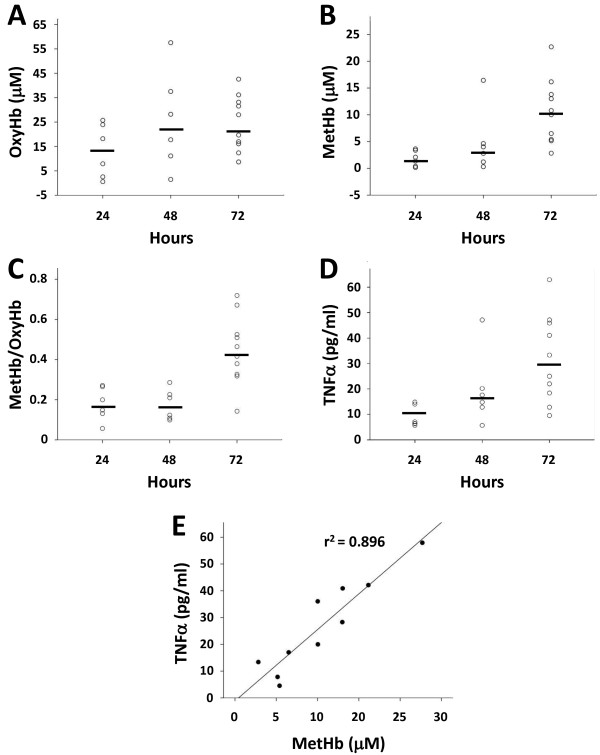
**High-frequency ultrasound of normal brain and cerebral IVH.** Coronal images obtained by high-frequency ultrasound displaying a normal brain with no IVH and cerebral IVH at six hours of age in preterm rabbit pups. Vertical ruler indicates 10 mm. IVH, intraventricular hemorrhage.

The animal protocols were approved by the Swedish Animal Ethics Committee in Lund. The experiments were performed using rabbit pups from a half-breed between New Zealand White and Lop, delivered at gestational day 29 (full gestational age 32 days). The pups were delivered by cesarean section after the does were anesthetized with i.v. propofol (5 mg/kg) and by local infiltration of the abdominal wall using lidocaine with adrenaline (10 mg/ml + 5 μl/ml, 20 to 30 ml). After delivery, the pups were dried vigorously, weighed, and placed in an infant incubator with a constant temperature of 36°C and 60% ambient humidity. At two to three hours of age, the pups were fed 1 ml of cat milk formula (KMR; PetAg Inc., Hampshire, IL, USA), and subsequently every 12 hours, increasing each meal by 0.5 ml. At two hours of age, 165 pups (from 25 litters) received an i.p. injection of 50% glycerol (6.5 g/kg, endotoxin-free as analyzed below) to induce IVH [10,19]. Using HFU imaging of the brain (VisualSonics Vevo 2100, VisualSonics Inc., Toronto, Canada, with a MS-550D 40MHz transducer) enabled accurate distinction of hemorrhagic extension [22] and was performed at 6, 24, 48, and 72 hours of age. At six hours of age, 110 pups (67%) displayed severe IVH (distended lateral ventricles filled by high-echogenic content with no parenchymal extension); 15 pups (9%) displayed a small/minor IVH (high-echogenic content within the lateral ventricles with no ventricular distention); and 40 pups (24%) displayed no signs of IVH. Images of pups with severe IVH or without signs of hemorrhage, as determined by HFU at six hours of age, are given in Figure 1. In this study, only pups with severe IVH (here referred to as the IVH group) and pups with no signs of cerebral hemorrhage (sham control group) were included (as determined by HFU at six hours of age). All control animals used in this study had received an i.p. injection of glycerol but did not exhibit any sign of IVH on cerebral ultrasound, thus ensuring that differences seen in IVH animals and control animals were not due to glycerol toxicity. In 22 rabbit pups with severe IVH, ultrasound-guided CSF sampling was performed at 24 (n = 6), 48 (n = 6) and 72 (n = 10) hours of age, as described previously [22]. Following CSF sampling, which was only performed once in each pup, the pups were euthanized and not further included in the study, that is, no further tissue or CSF sampling was performed on these pups. Accurate intraventricular CSF sampling from control pups was not possible due to the small, slit-like size of the ventricles in pups without hemorrhage. Immediately after sampling, the CSF samples were centrifuged (2,000 × g, 20°C, 10 minutes) to remove cells, and the supernatant was stored at -80°C until further analysis, as described below.

### Periventricular tissue collection and processing

Rabbit pups were euthanized at 24 (IVH = 6, sham control = 10) and 72 hours of age (IVH = 6, sham control = 17), and the brains were removed from the skulls and sectioned at the level of the midseptal nucleus. A 1-mm section around the periventricular zone was dissected, snap frozen, and stored at -80°C until further mRNA and protein analysis, as described below. Perfusion-fixation of brains was performed in an additional five rabbit pups (that is, not included in the groups for sampling of periventricular tissue, IVH = 3, sham control = 2) under isoflurane anesthesia at 72 hours of age by infusion of saline solution followed by 4% paraformaldehyde in PBS. Brains were then immersed in cryoprotective sucrose solution prior to cryosectioning for subsequent immunofluorescent analysis, as described below.

### Cerebrospinal fluid sampling from preterm infants

CSF was sampled serially from four preterm infants (gestational age at birth 25 to 28 weeks) 5 to 35 days after detection of IVH, by spinal tap or ventricular reservoir puncture. Immediately after sampling, the CSF was centrifuged (2,000 × g, 20°C for 10 minutes) and stored at -80°C until further analysis, as described below. The sampling was performed following written consent from the parents, and the study was approved by the ethical committee review board for studies in human subjects at Lund University.

### Preparation of oxy-, met-, and cyan-hemoglobin and hemin

Fetal oxyHb (HbF) was purified as previously described [23] from freshly drawn human umbilical cord blood. Briefly, red blood cells (RBCs) were isolated by centrifugation (1,200 × g, 10 minutes) and washed with excess PBS. The RBCs were then lysed by resuspension in hypotonic buffer (20 volumes H_2_O:1 volume PBS) on ice, and the membranes were separated from the cytosol by centrifugation (14,000 × g, 4°C, 20 minutes). The supernatant was applied to a DEAE-Sephadex A-50 (Amersham Biosciences AB, Uppsala, Sweden) column and separated using an increasing ion gradient. Fractions were collected and the absorbance measured to identify and determine the concentration of oxyHb. Human Hb was from Sigma Chemical Co. (St. Louis, MO, USA) and shown spectrophotometrically [23] to contain at least 70% to 80% metHb. This preparation is referred to here as metHb. Furthermore, metHb was also prepared by incubating the above described oxyHb solution at 37°C for 72 hours. The metHb concentration was quantified as described previously [23]. Hemin (ferriprotoporphyrin IX chloride) was purchased from Porphyrin Products Inc. (Logan, UT, USA), and a 10 mM stock solution was prepared using dimethyl sulfoxide (DMSO) (Sigma).

Cyan-Hb was prepared as described previously [24], by mixing HbF (1 mM) with KCN (10 mM) and incubating the mix for 10 minutes at 20°C. The solution was desalted on a Sephadex G-25 column (Amersham). Addition of cyanide to oxyHb locks the Fe^2+^ atom in its ferrous form, which disables spontaneous oxidation to metHb. All Hb derivatives (oxy-, met- (purchased and in-house prepared) and cyan-Hb) were purified from endotoxin contamination using the endotoxin removing product EndoTrap (Hyglos GmbH, Bernried, Germany) as described by the manufacturer. The absolute purity of all Hb derivatives (oxy-, met- (purchased and in-house prepared), cyanHb, and hemin) and the glycerol solution (used for i.p. injections) from contamination with endotoxin (0 EU/mg Hb/hemin) was determined using the QCL-1000™ Endpoint Chromogenic LAL Assay (Lonza, Basel, Switzerland ) and the lipopolysaccharide (LPS) ELISA Assay kit (Uscn Life Science Inc, Wuhan, China) as described by the manufacturers (Table 1).

**Table 1:** Endotoxin determination of Hb/hemin preparations

**Sample**	**Source**	**LAL assay before EndoTrap (EU/mg)**	**LAL assay after EndoTrap (EU/mg)**	**LPS ELISA after EndoTrap**
OxyHb	In-house purified	2.48	Not detectable	Not detectable
MetHb	In-house purified	Not tested	Not detectable	Not detectable
MetHb	Sigma	10.29	Not detectable	Not detectable
CyanHb	In-house purified	3.64	Not detectable	Not detectable
Hemin	Porphyrin Products	Not detectable	Not detectable	Not detectable

Determination of endotoxin levels in the Hb- and hemin preparations using an Endpoint Chromogenic LAL assay and a LPS ELISA assay as described in the Methods section. The endotoxin levels were determined both before and after purification of endotoxins using EndoTrap, as described in the Methods section. The endotoxin levels are determined as EU endotoxin per mg of Hb/hemin. *LPS*, lipopolysaccharide; *MetHb*, methemoglobin; *OxyHb*, oxyhemoglobin.

### Measurement of Hb metabolites in CSF

Hb metabolite concentrations were determined in CSF from preterm infants and rabbit pups using a spectrophotometric method described previously [23]. Briefly, samples were analyzed in a wavelength scan (250 to 700 nm) using a Beckman DU-800 spectrophotometer, and absorbance was specifically recorded for total protein (280 nm), oxyHb (540 and 577 nm), and metHb (630 nm). The concentration of oxyHb and metHb was calculated as described [23] and is displayed as Hb-tetramer concentration.

### Primary rabbit pup astrocyte cultures

Rabbit astroglial cell cultures were prepared from three-day-old healthy rabbit pups (not i.p. injected with glycerol and not used for any other sampling) according to a modified method described previously [25]. Briefly, after decapitation, the brain regions of interest were mechanically dissected and digested in trypsin/ethylenediaminetetraacetic acid (EDTA) solution. The tissue then was dissociated using a glass pasteur pipette and centrifuged; cells were resuspended in fresh culture medium and seeded in 75 cm^2^ flasks (cells from one brain/flask). Cells were grown in complete culture medium, which was changed every third day. After 10 days, cultures were shaken for one hour (250 rpm) to remove microglial cells, and astrocytes were resown in subcultures into appropriate culture dishes. When cells reached confluence (cultivation day six to eight), oxyHb, metHb, cyan-Hb and hemin (prepared immediately prior to the experiment, as described above) and a mixture of (NH_4_)Fe(SO_4_)_2_, hydrogen peroxide and ascorbate (the Fenton reaction) were added to the astrocyte cultures, and cells were incubated for one to four hours, as indicated in the figure legends. After incubation, culture medium was collected and cells harvested using Qiazol™ Lysis reagent (Qiagen Sciences, Germantown, MD, USA). Culture medium was analyzed for cell viability, total protein and TNFα protein concentration, and total RNA was extracted from cells to evaluate TNFα, IL-1β, TLR-4 and heme oxygenase (HO)-1 mRNA expression, as described below.

### Cell viability assay

The levels of lactate dehydrogenase (LDH) in astrocyte cell culture media were measured using the CytoTox 96® Non-Radioactive Cytotoxicity Assay (Promega, Madison, WI, USA) according to the instructions from the manufacturer.

### Total protein analysis

Total protein concentration in astrocyte cell culture media was determined using the Bradford protein assay, as described previously [24]. Albumin was used as a standard, and plotting the absorbance at 595 nm versus protein concentration generated a standard curve.

### RNA isolation and real-time PCR

Total RNA was isolated from the periventricular tissue and primary astrocytes using the acid guanidinium phenol–chloroform method supplied by Qiagen Sciences. The optical density (OD) ratio (260 nm/280 nm) of RNA was always higher than 1.8. Reverse transcription was performed according to the manufacturer’s instructions on 1 μg total RNA using an iScript^TM^ cDNA Synthesis Kit (Bio-Rad, Hercules, CA, USA). Real-time PCR was then used to quantify the TNFα, IL-1β, TLR-4 and HO-1 mRNA expression. Data were normalized to human glyceraldehyde-3-phosphate dehydrogenase (GAPDH). The fold-change values were calculated by normalizing against control samples from untreated animals or cells. Primers were designed accordingly: TNFα forward primer 5′-CTCCTACCCGAACAAGGTCA-3′, reverse primer 5′-CGGTCACCCTTCTCCAACT-3′; IL-1β forward primer 5′-AAGAAGAACCCGTCCTCTGCAACA-3′, reverse primer 5′-TCAGCTCATACGTGCCAGACAACA-3′; TLR-4 forward primer 5′-GAGCACCTGGACCTTTCAAATAAC-3′, reverse primer 5′-GAACTTCTAAACCACTCAGCCCTTG-′3; HO-1 forward primer 5′-GAGATTGAGCGCAACAAGGA-3′, reverse primer 5′-AGCGGTAGAGCTGCTTGAACT-′3; and GAPDH forward primer 5′-GAATCCACTGGCGTCTTCAC-3′, reverse primer 5′-CGTTGCTGACAATCTTGAGAGA-3′. Expression was analyzed using the Maxima SYBR Green qPCR Master Mix (Thermo Scientific Fermentas, Göteborg, Sweden). Amplification was performed at the respective adequate temperature for 40 cycles in an iCycler Thermal Cycler (Bio-Rad) and data analyzed using iCycler iQ Optical System Software (Bio-Rad).

### TNFα ELISA

The concentrations of TNFα in CSF (preterm infants and rabbit pups), in periventricular tissue from rabbit pups and in astrocyte cell culture media were determined using the Human (CSF preterm infants) and Rabbit TNFα DuoSet ELISA Development kits from R&D Systems, Abingdon, UK. The analysis was performed according to the instructions from the manufacturer.

### Immunofluorescence

Brain sections (40 μm) from paraformaldehyde-perfused animals were washed in PBS, blocked with 2% normal horse serum and incubated with a polyclonal goat anti-TNFα (1:50, Santa Cruz Biotechnology, Santa Cruz, CA, USA). After overnight incubation at 4°C, sections were incubated with a donkey anti-goat biotinylated secondary antibody (diluted at 1:200, Jackson ImmunoResearch Laboratories, West Grove, PA, USA). Sections were further incubated with an Alexa-488 streptavidin conjugate (1:200, Invitrogen, Stockholm, Sweden) and a Cy3-conjugated monoclonal anti-glial fibrillary acidic protein (GFAP) antibody (1:500, Sigma-Aldrich, Stockholm, Sweden). Fluorescent signals were visualized using a confocal microscopy system (LSM510, Zeiss, Oberkochen, Germany ).

### Statistics

Pair-wise comparisons between unrelated groups were performed with the Students *t*-test or the Mann–Whitney *U* test as appropriate. Comparisons between multiple groups were performed by analysis of variance (ANOVA) with *post hoc* Bonferroni correction. Correlations were assessed by linear regression analysis. *P* values <0.05 were considered significant.

## Results

### Extracellular Hb metabolites and TNFα in CSF following IVH

**Figure 2:**
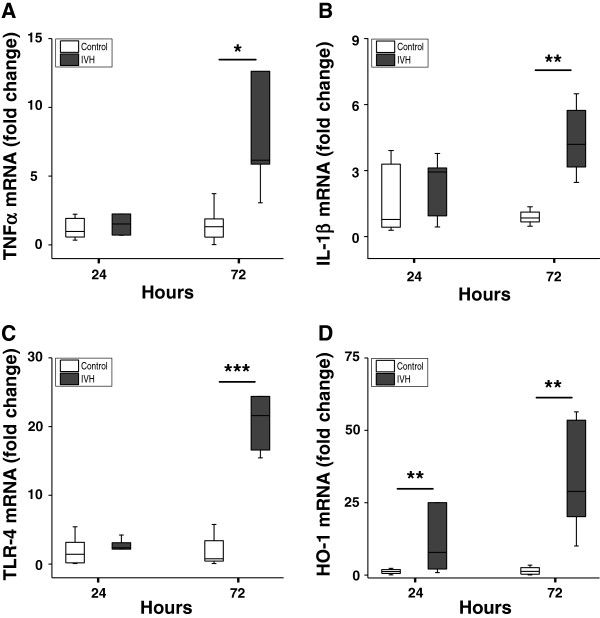
**Hb metabolites and TNFα in CSF from rabbit pups following IVH.** OxyHb **(A)**, metHb **(B)**, and TNFα **(D)** were quantified in intraventricular CSF at 24 (n = 6), 48 (n = 6), and 72 (n = 10) hours, as described in the Methods section, and the ratio of oxyHb/metHb was calculated **(C)**. Horizontal lines depict median values. Median values of metHb and TNFα were increased at 72 hours as compared to corresponding values at 24 and 48 hours (*P* <0.01, Mann–Whitney U). The correlation between TNFα and metHb **(E)** at 72 hours was determined by linear regression analysis (r^2^ = 0.896, *P* <0.001). CSF, cerebrospinal fluid; Hb, hemoglobin; IVH, intraventricular hemorrhage; metHb, methemoglobin; oxyHb, oxyhemoglobin.

Intraventricular CSF concentrations of extracellular oxyHb, metHb, and TNFα in rabbit pups with IVH were assessed at 24, 48, and 72 hours of age (Figure 2). The median concentration of oxyHb did not change significantly over time (Figure 2A) whereas that of metHb was significantly increased at 72 hours compared to values at 24 and 48 hours (Figure 2B), illustrated by the increasing ratio of metHb/oxyHb over time (Figure 2C). Similarly to metHb, the concentration of TNFα increased significantly over time and was highest at 72 hours as compared to 24 and 48 hours (Figure 2D). Furthermore, concentrations of metHb and TNFα exhibited a strong positive correlation at 72 hours (r^2^ = 0.896, *P* <0.001, Figure 2E) whereas no correlation was observed between oxyHb and TNFα (not shown).

### Inflammation in periventricular brain tissue following IVH

**Figure 3:**
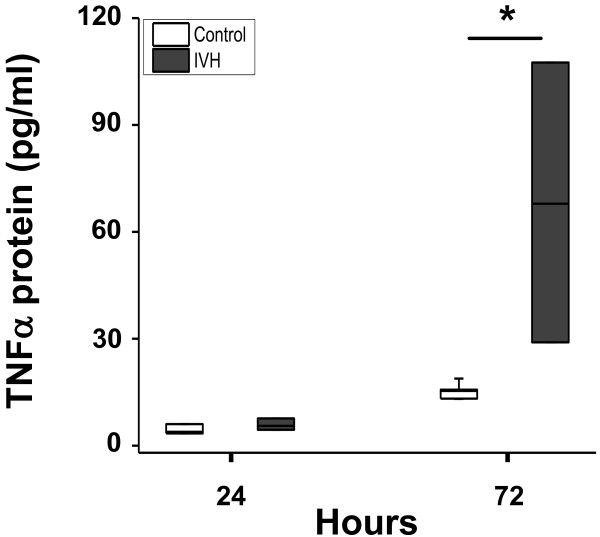
**mRNA expression in periventricular brain tissue.** mRNA expression of TNFα **(A)**, IL-1β **(B)**, TLR-4 **(C)** and HO-1 **(D)** in periventricular brain tissue of preterm rabbit pups with IVH (dark shaded bars; n = 6 at 24 hours and n = 6 at 72 hours) and in control pups (white bars; n = 10 at 24 hours and n = 17 at 72 hours). Expression of mRNA was determined using real-time PCR, as described in the Methods section, and levels of TNFα, IL-1β, TLR-4 and HO-1, respectively, were normalized against those of GAPDH and are given as fold change. The fold-change values were calculated by normalizing against control samples from untreated animals. Results are presented as box plots displaying medians and 25th and 75th percentiles. Differences between IVH versus control at 24 and 72 hours, respectively, were analyzed using the Mann–Whitney *U* test. * *P* <0.05, ** <0.01, *** <0.001. GAPDH, glyceraldehyde-3-phosphate dehydrogenase; HO-1, heme oxygenase; IVH, intraventricular hemorrhage; TLR-4, Toll-like receptor-4.

**Figure 4:**
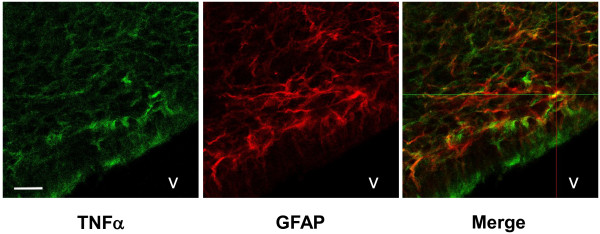
**TNFα protein in periventricular brain tissue.** Determination of TNFα protein concentration in periventricular brain tissue of preterm rabbit pups with IVH (dark shaded bars; n = 6 at 24 hours and n = 6 at 72 hours) and in control pups (white bars; n = 6 at 24 hours and n = 6 at 72 hours), using ELISA, as described in the Methods section. Results are presented as box plots displaying medians and 25th and 75th percentiles. The difference between IVH versus control at 72 hours was analyzed using the Mann–Whitney *U* test. * *P* <0.05. IVH, intraventricular hemorrhage.

**Figure 5:**
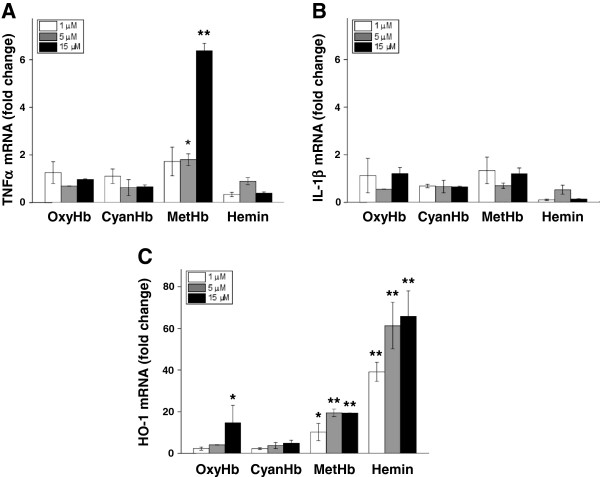
**TNFα and GFAP immunofluorescence following IVH.** Immunofluorescence of rabbit pup brain sections at 72 hours following IVH was performed as described in the Methods section. Sections were stained against TNFα (green, AF-488) and GFAP (red, Cy3) and displayed a co-staining for TNFα in GFAP-positive astrocytes. Scale bar, 20 μm. V, ventricle. GFAP, glial fibrillary acidic protein; IVH, intraventricular hemorrhage.

The median levels of mRNA expression for TNFα, IL-1β, TLR-4 and HO-1 were increased in periventricular brain tissue in rabbit pups with IVH as compared to control pups (Figure 3). The increases in TNFα, IL-1β and TLR-4 mRNA were significant (*P* <0.05, TNFα; *P* <0.01, IL-1β; *P* <0.001, TLR-4) at 72 hours but not at 24 hours. Correspondingly, the median level of TNFα protein was increased at 72 hours in IVH pups (67.9 pg/mg total protein) as compared to control pups (15.4 pg/mg total protein; *P* = 0.02) but not at 24 hours (Figure 4). In addition, immunohistochemistry showed positive TNFα staining in periventricular brain tissue in rabbit pups with IVH at 72 hours (Figure 5). Counter staining for astrocyte activation (GFAP-positive staining) revealed the presence of TNFα in GFAP-positive astrocytes but also in non–GFAP-positive cells.

### Exposure of astrocyte cultures to Hb metabolites and ROS

**Figure 6:**
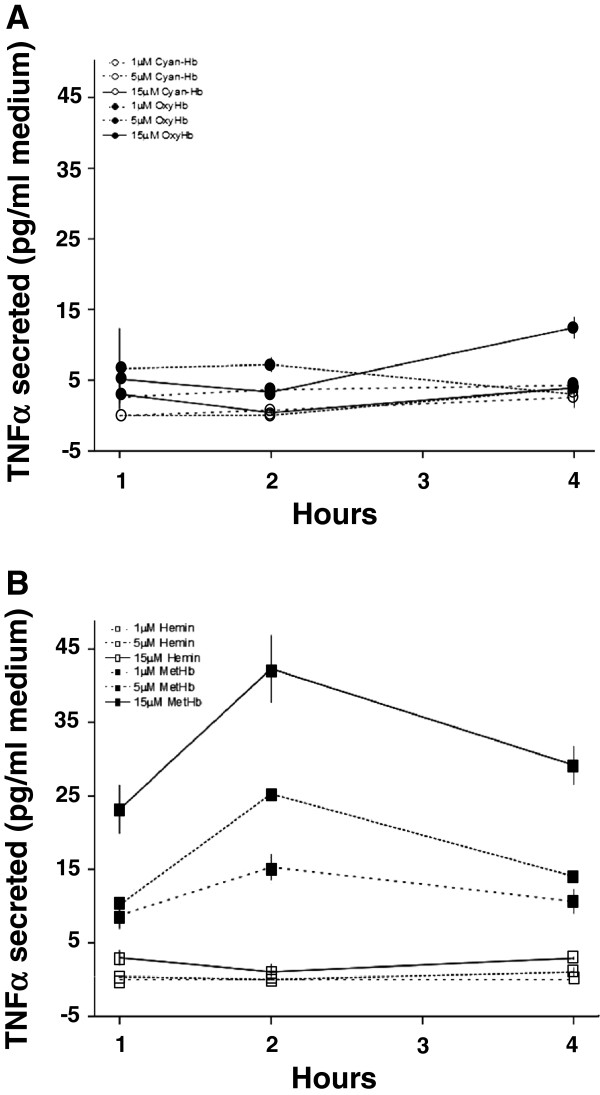
**Hb metabolite–induced TNFα, IL-1β, and HO-1 mRNA expression in astrocyte cell cultures.** mRNA expression of TNFα **(A)**, IL-1β **(B)** and HO-1 **(C)** in primary rabbit astrocyte cell cultures exposed to oxyHb, metHb, cyan-Hb, and hemin for four hours at concentrations of 1 μM (white bars), 5 μM (light shaded bars) and 15 μM (dark shaded bars), was determined using real-time PCR, as described in the Methods section. The mRNA expression of TNFα, IL-1β and HO-1 was normalized against GAPDH and is given as fold change. The fold-change values were calculated by normalizing against control samples from untreated cells. Results are from triplicate experiments and presented as mean ± SEM. Differences between the respective exposures and control conditions were analyzed using Mann–Whitney U. * *P* <0.05, ** <0.01. GAPDH, glyceraldehyde-3-phosphate dehydrogenase; Hb, hemoglobin; HO-1, heme oxygenase; metHb, methemoglobin; oxyHb, oxyhemoglobin; SEM, standard error of the mean.

**Figure 7:**
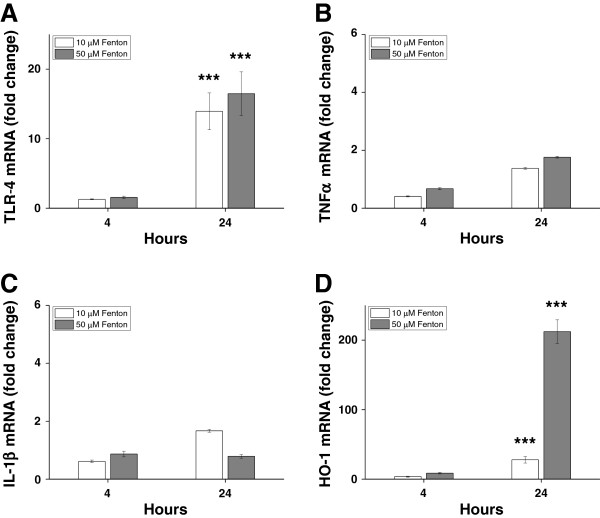
**Hb metabolite–induced TNFα protein secretion in astrocyte cell cultures.** Determination of TNFα protein concentration in culture medium of primary rabbit astrocyte cell cultures, exposed for one to four hours to oxyHb, cyan-Hb, metHb and hemin at 1, 5 and 15 μM, respectively, using ELISA, as described in the Methods section. Exposure to oxyHb (filled circles) and cyan-Hb (open circles) is illustrated in panel **A** and metHb (filled squares) and hemin (open squares) in panel **B**. Continuous line = 15 μM; dotted line = 5 μM; hatched line = 1 μM. Results are from triplicate experiments and are presented as mean ± SEM. MetHb at 1, 5 and 15 μM versus control, all *P* <0.01 (ANOVA for repeated measures). ANOVA, analysis of variance; Hb, hemoglobin; metHb, methemoglobin; oxyHb, oxyhemoglobin; SEM, standard error of the mean.

The strong correlation observed between extracellular metHb and TNFα in intraventricular CSF suggested that metHb or metabolites of Hb may be causal initiators of inflammation following IVH. These relationships were evaluated in primary rabbit astrocyte cell cultures. Exposing the astrocytes to oxyHb, cyan-Hb, metHb or hemin at concentrations of 1 to 15 μM led to dose-dependent HO-1 mRNA expression with each reagent as compared to controls (Figure 6C). TNFα mRNA expression increased dose-dependently only after exposure to metHb but to not oxyHb, cyan-Hb or hemin (Figure 6A) whereas no reagent affected IL-1β mRNA expression as compared to control cultures (Figure 6B). Furthermore, exposure to metHb caused a significant, dose-dependent increase in TNFα protein concentrations in culture medium at one, two, and four hours of exposure, with the highest levels noted after two hours (Figure 7B). Exposure to oxyHb at corresponding concentrations resulted in a small but significant increase in levels of TNFα protein (Figure 7A). This increase was not observed following cyan-Hb exposure, strongly indicating that conversion of oxyHb to metHb is necessary for TNFα induction. Additionally, exposure to free hemin did not cause a measurable increase in TNFα protein in culture medium (Figure 7B). In order to assure that the results obtained with metHb were not caused by endotoxin contamination in the purchased metHb derivative, confirming experiments were performed. Endotoxin-free oxyHb (in-house purified as described in the Methods section) was oxidized to metHb under sterile conditions (as described in the Methods section), and astrocytes were exposed to oxyHb and metHb as described above. Analysis of TNFα mRNA from astrocytes [see Additional file 1: Figure S1A] and of TNFα protein [see Additional file 1: Figure S1B] in culture medium displayed very similar results as those obtained with purchased and purified metHb.

**Figure 8:**
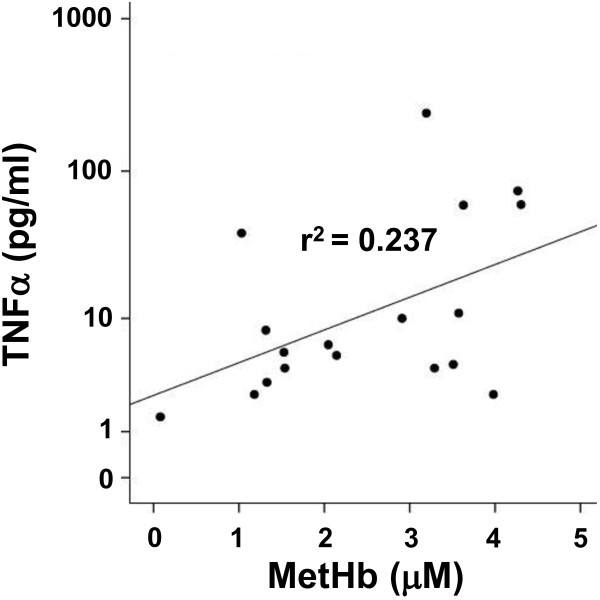
**Fenton reaction–induced TLR-4, TNFα, IL-1β and HO-1 mRNA expression in astrocyte cell cultures.** Primary rabbit astrocyte cell cultures were exposed to a mixture of (NH_4_)Fe(SO_4_)_2_, hydrogen peroxide and ascorbate (the Fenton reaction) for four hours at concentrations of 10 μM (NH_4_)Fe(SO_4_)_2_ + 100 μM ascorbate + 20 μM H_2_O_2_ (white bars) or 50 μM (NH_4_)Fe(SO_4_)_2_, 500 μM ascorbate + 100 μM H_2_O_2_ (shaded bars). mRNA expression of TLR-4 **(A)**, TNFα **(B)**, IL-1β **(C)** and HO-1 **(D)** was determined using real-time PCR, as described in the Methods section. The mRNA expression of TLR-4, TNFα, IL-1β and HO-1 was normalized against GAPDH and is given as fold change. The fold-change values were calculated by normalizing against control samples from untreated cells. Results are from triplicate experiments and presented as mean ± SEM. Differences between the respective exposures and control conditions were analyzed using Mann–Whitney U. *** *P* <0.001. GAPDH, glyceraldehyde-3-phosphate dehydrogenase; HO-1, heme oxygenase; SEM, standard error of the mean; TLR-4, Toll-like receptor-4.

Exposure to the Fenton reaction, (that is, a mixture of Fe^3+^, ascorbate and hydrogen peroxide) was performed to evaluate the pro-inflammatory effect of the hydroxyl radical. Exposure to the Fenton reaction–generated hydroxyl radicals caused a highly significant upregulation of TLR-4 and HO-1 mRNA expression, but not of TNFα or IL-1β (Figure 8). Furthermore, no increase in TNFα protein concentrations was observed in culture medium of cells exposed to the Fenton-reaction mixture, as compared to control cultures (not shown).

The cytotoxicity assay (LDH) showed that exposure of astrocytes to oxyHb, metHb, and hemin for four hours at concentrations up to 30 μM resulted in a low but similar degree of cell death for metHb (approximately 13%) and hemin (approximately 20%) but significantly less for oxyHb (approximately 2%; *P* = 0.003).

### Extracellular Hb and TNFα in CSF of preterm infants with IVH

**Figure 9:**
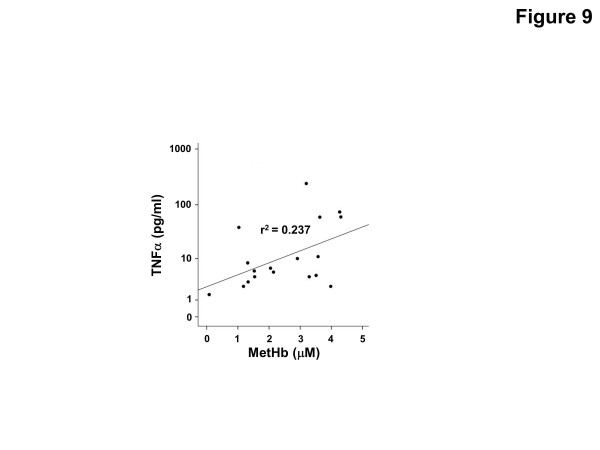
**Hb metabolites and TNFα in CSF from preterm infants following IVH.** Levels of metHb and TNFα from CSF obtained in serial samples from four preterm infants with IVH were determined as described in the Methods section. The correlation between TNFα and metHb was determined by linear regression analysis (r^2^ = 0.237, *P* = 0.01). CSF, cerebrospinal fluid; Hb, hemoglobin; IVH, intraventricular hemorrhage; metHb, methemoglobin.

Following IVH, serial CSF samples from four preterm infants were collected (as described above), and the concentrations of cell-free oxyHb, metHb and TNFα were determined. A positive correlation was observed between concentrations of metHb and TNFα (r^2^ = 0.237, *P* = 0.01) (Figure 9), but no significant correlation was identified between those of oxyHb and TNFα (r^2^ = 0.01, *P* = 0.7) (not shown).

## Discussion

Our results demonstrate for the first time that extracellular metHb, an oxidized metabolite of Hb, can induce an inflammatory response following preterm IVH. We show that intraventricular CSF levels of metHb accumulate during the first days after IVH and that levels of metHb are highly correlated with those of the key pro-inflammatory mediator TNFα. In fact, following IVH *in vivo*, extracellular metHb reaches levels in the intraventricular space by three days that can induce TNFα *in vitro*. We propose that induction of TNFα by metHb is an early upstream event in the development of periventricular brain damage.

Previous studies evaluating the effects of blood components in brain hemorrhage have largely relied on injection of autologous blood or blood components [21]. Glycerol-induced hyperosmolarity in the preterm rabbit pup model causes an increased transmural pressure gradient, vessel rupture and leakage of blood from the intravascular to the intraventricular space [26]. This sequence of events mirrors closely the pathophysiology known to occur in IVH in the preterm human infant. By using HFU, the size and distribution of hemorrhage could be strictly defined throughout the study, and, thus, only pups with a large hemorrhage restricted to the intraventricular space were included. This distinction is important when considering the primary site of the initiated inflammatory response and a hypothesized secondary extension to adjacent structures not initially affected by extravasated blood.

We found that levels of extracellular oxyHb were relatively unchanged in intraventricular CSF during the first three days following hemorrhage, suggesting a constant rate of hemolysis and a proportionate degradation into Hb metabolites, including auto-oxidation to metHb. In contrast to oxyHb, our data clearly show that the intraventricular levels of extracellular metHb increased over time. This finding is supported by previous clinical and experimental studies in adult hemorrhages. For example, serial evaluation using magnetic resonance imaging in adult humans with ICH and subarachnoid hemorrhage (SAH) and in animal models of ICH indicated that extracellular metHb is detectable at three days after onset of hemorrhage [27]. These findings coincide with the finding that three days after injection of either whole blood or packed erythrocytes in adult rats, maximal brain edema formation is observed, an effect attributed to cell-free Hb metabolites [28]. To our knowledge, quantitative *in vivo* characterization of extracellular metHb accumulation and its correlation to the inflammatory response has not previously been performed following preterm cerebral IVH.

Our findings indicate that metHb, but not oxyHb or hemin, induces TNFα transcription and protein secretion in astrocyte cell cultures. Future studies will evaluate if the TNFα-inducing capacity of metHb arises from ligand–receptor interaction or from the inherent redox properties of metHb. Liu *et al*. [16] showed that metHb induces NF-κB-mediated upregulation of IL-6, IL-8, and E-selectin in endothelial cells with no corresponding effect of either oxyHb or heme exposure. These authors also found that the pro-inflammatory effect of metHb did not depend on digestion by HO-1 or on the presence of free iron. Conversely, we found no effect on TNFα induction by exposure to either hemin or to the Fenton-induced hydroxyl radical, that is, the principal iron-mediated ROS.

At 72 hours after IVH, we did identify a temporal coincidence between the increased TNFα and IL-1β protein and mRNA levels in periventricular tissue and the increased TNFα in CSF. The cellular source of TNFα in the intraventricular space remains to be characterized, but it is most likely secreted from macrophages and neutrophils recruited to the intraventricular space. Previous work using the rabbit pup model of IVH has shown that microglial activation, astrocytic gliosis and neutrophil infiltration in periventricular tissue reach a clear peak at 72 hours following IVH [10]. It was outside the scope of the current study to investigate to what extent extracellular Hb metabolites penetrate the ependymal lining into the periventricular tissue, a prerequisite for periventricular microglial or astrocytic activation by metHb. However, this endpoint is currently being investigated in ongoing studies.

Our *in vitro* and *in vivo* results have largely focused on the induced expression and synthesis of the key pro-inflammatory mediator TNFα. TNFα signaling is involved in multiple aspects of inflammatory brain injury and results in two major responses: apoptosis and inflammation [29]. TNFα knockout mice and antisense oligodeoxynucleotide-treated adult rats with cerebral hemorrhage exhibit diminished cell death and inflammation compared to controls [30,31]. A more recent study showed that intracerebroventricular treatment with a TNFα inhibitor is associated with decreased periventricular cell death, gliosis and neuronal degeneration in the rabbit pup model of preterm IVH [12].

Inflammatory cytokines have been described as being rapidly upregulated in animal models of brain hemorrhage and ischemia, with an early peak at 24 to 48 hours following the insult [32]. In our study, we did not investigate the inflammatory response *in vivo* during the first 24 hours following hemorrhage because reproducible CSF sampling is difficult prior to an established ventricular dilatation. Thus, it is not known if there is an early inflammatory response preceding the increased inflammatory response reported, that is, a biphasic response.

The predominant pathway for Hb clearance is the CD163–haptoglobin–hemoglobin system. A recent study in human adults showed that the resting state capacity of the intrathecal CD163–haptoglobin–hemoglobin clearing system is 50,000-fold lower than that of the circulation and that this system is quickly saturated following SAH with a residual inability to deal effectively with extracellular Hb [33]. Furthermore, the normal circulating levels of haptoglobin are reported to be very low in preterm infants, indicating a heightened vulnerability to extracellular Hb [34,35]. To date, the concentration of intrathecal haptoglobin in preterm infants has not been determined, but it is reasonable to assume that it is extremely low because systemic haptoglobin contributes to intrathecal haptoglobin levels. In our study, we observed high levels of extracellular oxyHb and metHb in CSF from preterm rabbit pups following IVH, similar to those detected in CSF from preterm human infants with IVH. These high levels of extracellular Hb metabolites, which can induce a robust pro-inflammatory response both *in vivo* and *in vitro*, indicate the insufficiency of the endogenous intrathecal clearance systems within the immature brain.

In addition, we provide the first evidence that IVH in the immature brain is associated with an upregulation of TLR-4 mRNA, manifested in periventricular brain tissue at 72 hours after IVH. Activation of TLR-4 has been assigned a prominent role in adult ICH as a mediator of pro-inflammation, cerebral edema and neurological deficit. In animal models of adult ICH, TLR-4 protein and mRNA expression peak at three days after hemorrhage and can be detected in neurons, astrocytes and, predominantly, in microglia [36]. We did not observe increased TLR-4 mRNA expression after exposing astrocytes to Hb metabolites whereas exposure to the Fenton-generated hydroxyl radical indeed resulted in increased mRNA expression. The absence of TLR-4 upregulation following exposure to Hb metabolites does not preclude that the TNFα-inducing effect of metHb is mediated by signaling through this receptor. Indeed, previous studies have shown that astrocytes can activate TLR-4, triggering intracellular pathways that lead to a pro-inflammatory environment including TNFα synthesis [37]. Furthermore, others have shown that ROS play an interactive role in TLR-4 activation, mainly by modifying intracellular pathways downstream of TLR-4. A recent study found that heme-induced microglial activation *in vitro* and the pro-inflammatory cytokine response *in vivo* were completely blocked in TLR-4 knockout mice [18].

## Conclusions

Our results show that extracellular metHb accumulates in the intraventricular space during the first 72 hours following preterm IVH and induces expression of the pro-inflammatory cytokine TNFα. Signaling by TNFα is a key event resulting in inflammation, apoptosis, and subsequent periventricular brain damage. Our data suggest that there is a defined time window after onset of hemorrhage that might be accessible for therapeutic intervention. We propose that treatment aimed at reducing intraventricular accumulation of Hb and Hb metabolites has the potential to limit inflammation and tissue damage following IVH.

## Abbreviations

ANOVA: Analysis of variance; CSF: Cerebrospinal fluid; ELISA: Enzyme-linked immunosorbent assay; Fe3+: Ferric; Fe2+: Ferrous; Fe4+: Ferryl; HbF: Fetal oxyHb; GFAP: Glial fibrillary acidic protein; GAPDH: Glyceraldehyde-3-phosphate dehydrogenase; HO: Heme oxygenase; Hb: Hemoglobin; HFU: High-frequency ultrasound; ICH: Intracranial hemorrhage; IL: Interleukin; IVH: Intraventricular hemorrhage; LDH: Lactate dehydrogenase; LPS: Lipopolysaccharide; metHb: Methemoglobin; OD: Optical density; oxyHb: Oxyhemoglobin; PBS: Phosphate-buffered saline; PCR: Polymerase chain reaction; RBC: Red blood cell; ROS: Reactive oxygen species; SAH: Subarachnoid hemorrhage; TNF: Tumor necrosis factor; TNFR: Tumor necrosis factor receptor; TLR: Toll-like receptor.

## Competing interests

The authors declare that they have no competing interests.

## Authors’ contributions

MG, SS, SRH, BÅ and DL planned and designed the experiments. MG, SS, KR, MC and DL performed the experiments. MG, SS, KR, BÅ and DL performed data analysis and interpreted the results. MG, SS, KR, SRH, MC, BÅ and DL wrote and revised the manuscript. MG, SS and DL submitted the manuscript. All authors read and approved the final manuscript.

## Supplementary Material

Additional file 1: Figure S1 **Hb metabolite–induced TNFα mRNA expression and protein secretion in astrocyte cell cultures. A.** mRNA expression of TNFα in primary rabbit astrocyte cell cultures, exposed to oxyHb and metHb for four hours at concentrations of 1 μM (white bars), 5 μM (light shaded bars), and 15 μM (dark shaded bars), was determined using real-time PCR, as described in the Methods section. The mRNA expression of TNFα was normalized against GAPDH and is given as fold change. The fold-change values were calculated by normalizing against control samples from untreated cells. Results are from triplicate experiments and presented as mean ± SEM. Differences between the respective exposures and control conditions were analyzed using Mann–Whitney U. ** *P* <0.01. **B.** Determination of TNFα protein concentration in culture medium of primary rabbit astrocyte cell cultures, exposed for one to four hours to oxyHb (open squares) and metHb (filled squares) at 1, 5, and 15 μM, respectively, using ELISA, as described in the Methods section. Continuous line = 15 μM; dotted line = 5 μM; hatched line = 1 μM. Results are from triplicate experiments and are presented as mean ± SEM. MetHb at 1, 5, and 15 μM versus control, all *P* <0.01 (ANOVA for repeated measures). Click here for file
