# Supplementary figures and images for: Hemoglobin induces inflammation after preterm intraventricular hemorrhage by methemoglobin formation
Source: J Neuroinflammation. 2013 Aug 6;10:100. doi: 10.1186/1742-2094-10-100 (PMC3750409; doi:10.1186/1742-2094-10-100)

## Slide 1
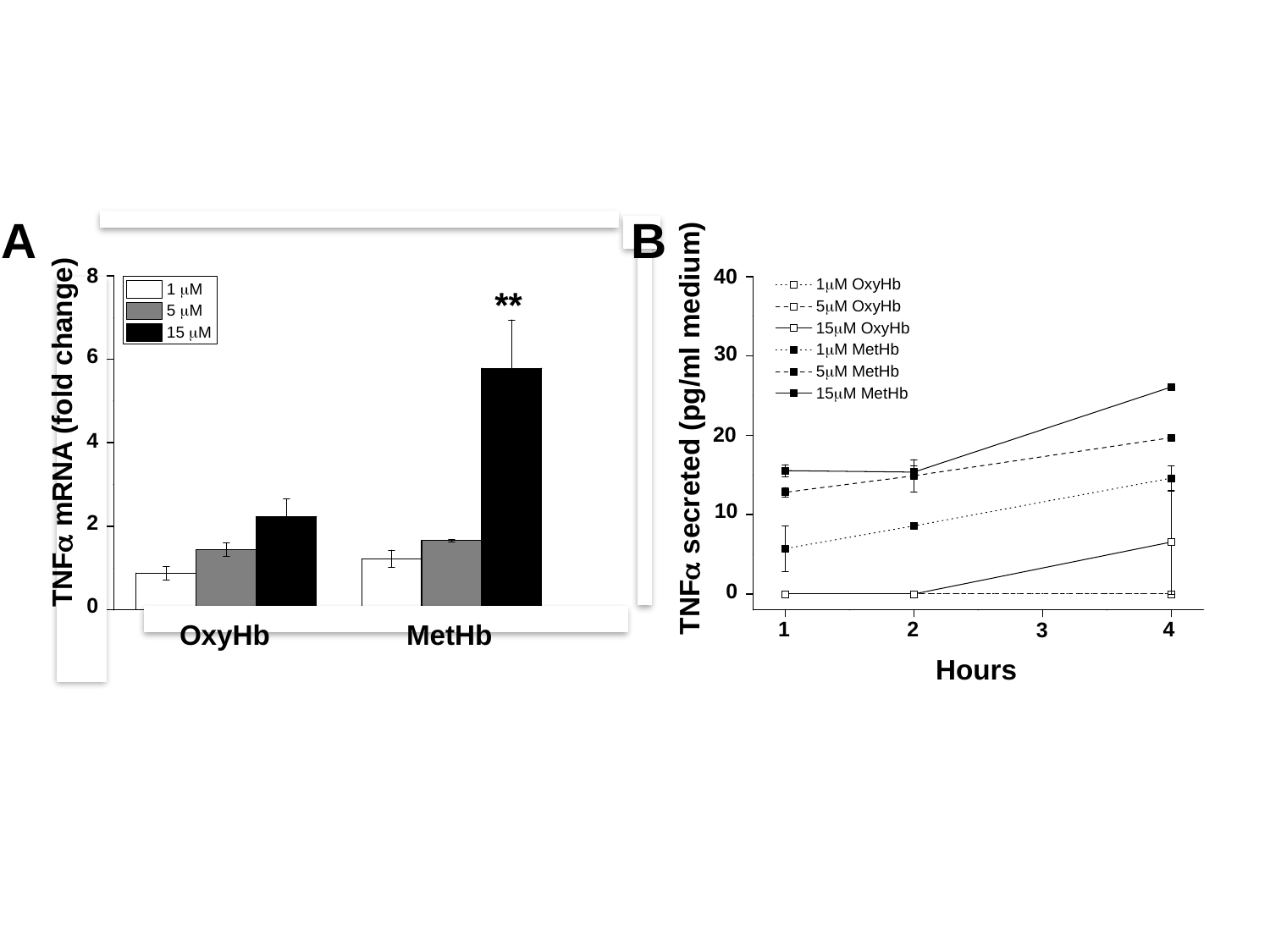

A
B
8
40
**
30
6
TNFα secreted (pg/ml medium)
TNFα mRNA (fold change)
20
4
10
2
0
0
1
2
4
3
OxyHb
MetHb
Hours

Supplement: Additional file 1: Figure S1 — Hb metabolite–induced TNFα mRNA expression and protein secretion in astrocyte cell cultures. A. mRNA expression of TNFα in primary rabbit astrocyte cell cultures, exposed to oxyHb and metHb for four hours at concentrations of 1 μM (white bars), 5 μM (light shaded bars), and 15 μM (dark shaded bars), was determined using real-time PCR, as described in the Methods section. The mRNA expression of TNFα was normalized against GAPDH and is given as fold change. The fold-change values were calculated by normalizing against control samples from untreated cells. Results are from triplicate experiments and presented as mean ± SEM. Differences between the respective exposures and control conditions were analyzed using Mann–Whitney U. ** P <0.01. B. Determination of TNFα protein concentration in culture medium of primary rabbit astrocyte cell cultures, exposed for one to four hours to oxyHb (open squares) and metHb (filled squares) at 1, 5, and 15 μM, respectively, using ELISA, as described in the Methods section. Continuous line = 15 μM; dotted line = 5 μM; hatched line = 1 μM. Results are from triplicate experiments and are presented as mean ± SEM. MetHb at 1, 5, and 15 μM versus control, all P <0.01 (ANOVA for repeated measures). [file 1742-2094-10-100-S1.pptx]
